# Supplementary material for: The N-Terminal Domain of Tailspike Depolymerases Affects the Replication Efficiency of Synthetic Klebsiella Phages
Source: Int J Mol Sci. 2025 Nov 22;26(23):11297. doi: 10.3390/ijms262311297 (PMC12692247; doi:10.3390/ijms262311297)
Supplement: Supplementary file 1 [file ijms-26-11297-s001.zip › ijms-3933188-supplementary.pdf]

Supplementary materials

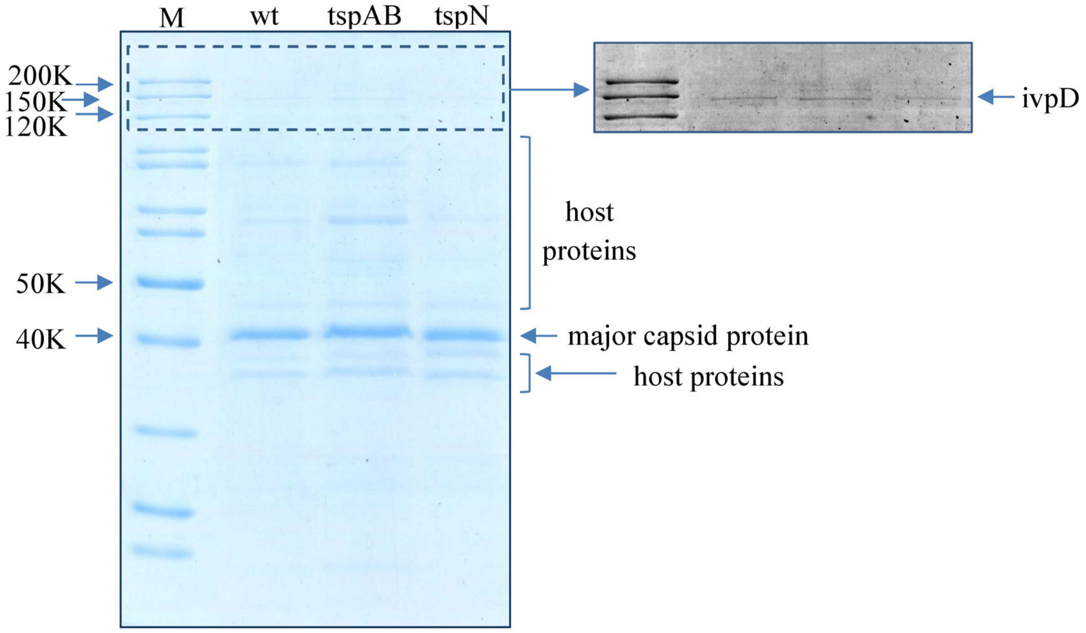

Figure S1. Equalization of Phage Particle Concentration in Phage Samples

Stained 12% (w/v) polyacrylamide gel after denaturing protein electrophoresis (SDS-PAGE), 1.5 µl of phage samples applied. M – protein ladder #26614 (Thermo Fisher Scientific); “wt”, “tspAB” and “tspN” denote phages KP192, KP195_tspAB192 and KP195_tspN$_{195}$AB192, respectively. “ivpD” denotes additional reference band corresponding to the internal virion protein D, MW = 143 kDa (shown on an image fragment subjected to additional software contrasting).

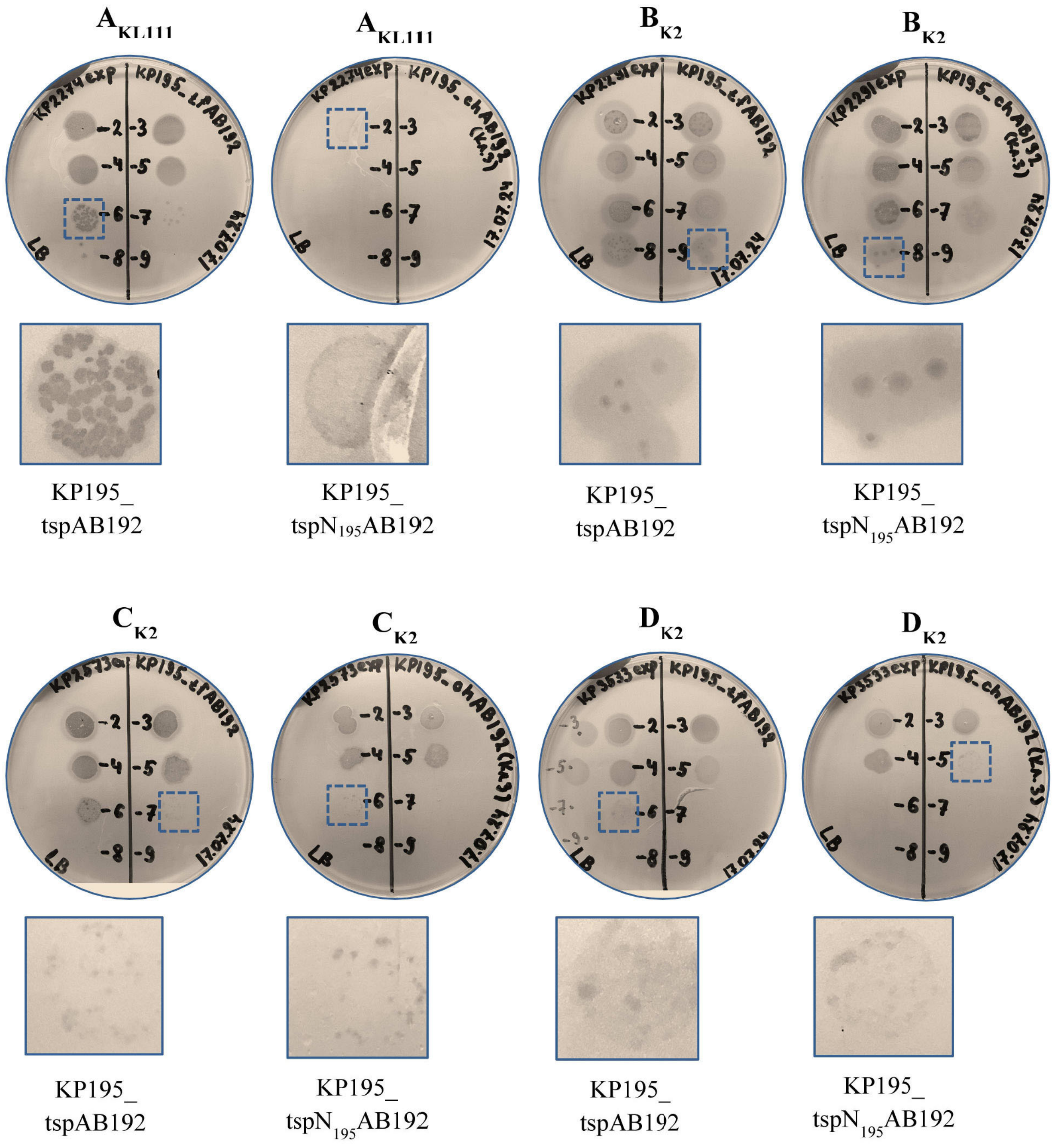

Figure S2. Efficiency of plating (EOP) and plaque morphology of phages KP195_tspAB192 and KP195_tspN$_{195}$AB192 on *Klebsiella* strains A$_{KL111}$, B$_{K2}$, C$_{K2}$, and D$_{K2}$

Serial tenfold dilutions (5 µl) of each phage were spotted onto a top agar lawn of exponentially growing *Klebsiella* host strains. Phage stock concentrations were 4.6 × 10^{11} PCLU/mL. Assays were performed on 90 mm Petri dishes; representative 10 × 10 mm areas are shown at higher magnification.

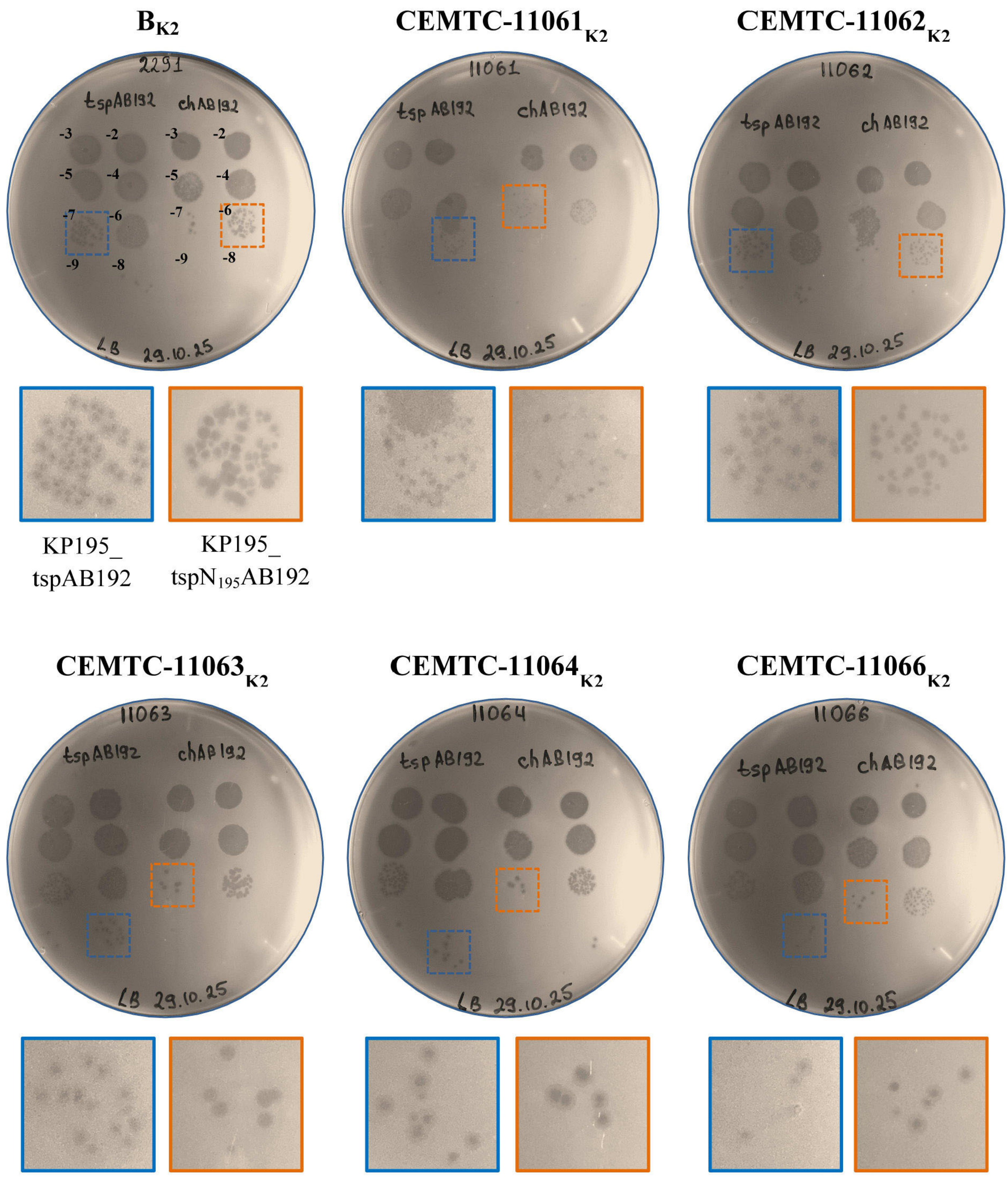

Figure S3. Efficiency of plating (EOP) and plaque morphology of phages KP195_tspAB192 and KP195_tspN$_{195}$AB192 on *Klebsiella* strains CEMTC-11061$_{K2}$, CEMTC-11062$_{K2}$, CEMTC-11063$_{K2}$, CEMTC-11064$_{K2}$, and CEMTC-11066$_{K2}$

Serial tenfold dilutions (5 µl) of each phage were spotted onto a top agar lawn of exponentially growing *Klebsiella* host strains. Phage stock concentrations were 4.6 × 10^{11} PCLU/mL. Assays were performed on 90 mm Petri dishes; representative 10 × 10 mm areas are shown at higher magnification. B$_{K2}$ strain was used as a control.

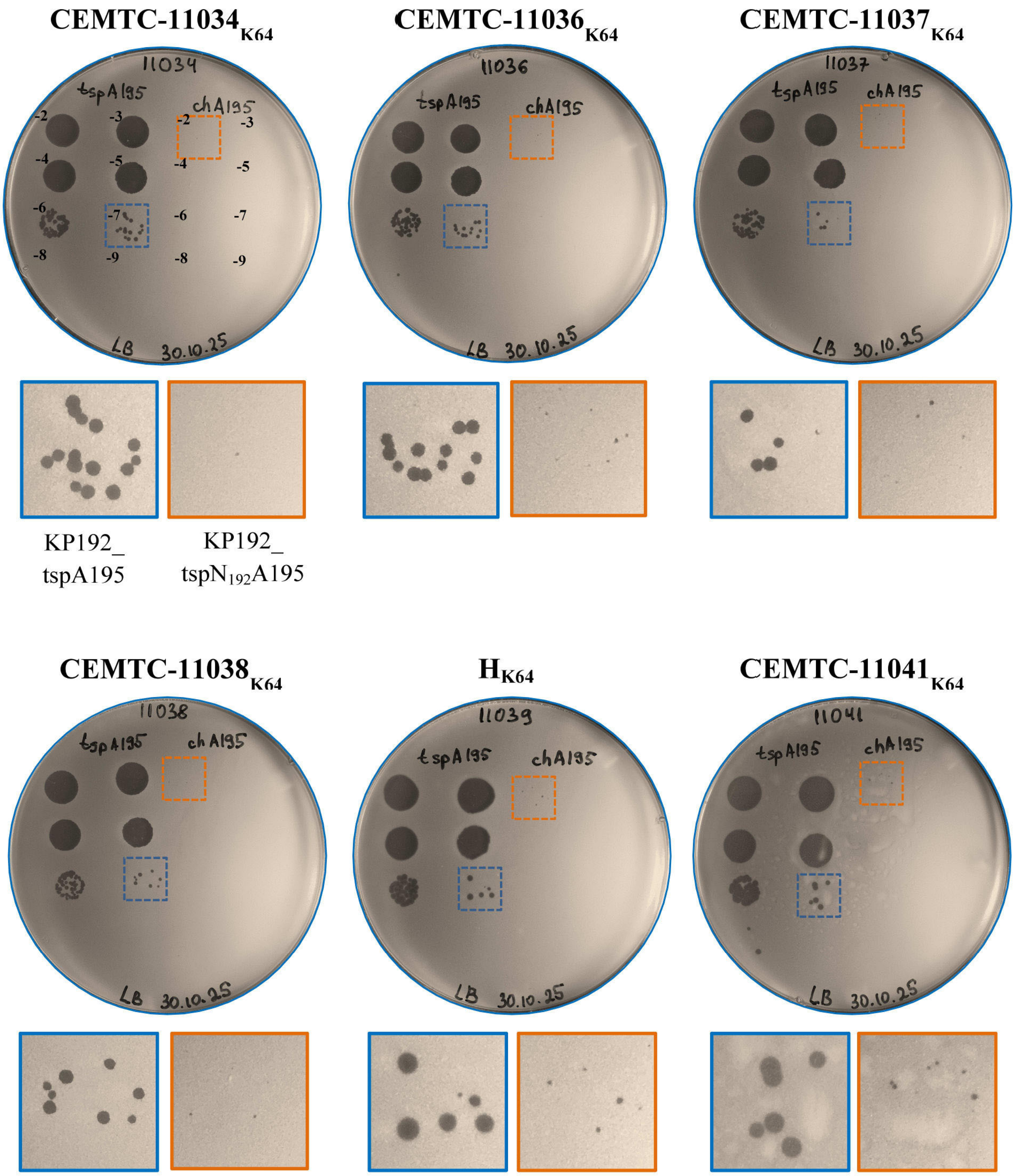

Figure S4. Efficiency of plating (EOP) and plaque morphology of phages KP192_tspA195 and KP192_tspN$_{192}$A195 on *Klebsiella* strains CEMTC-11034$_{K64}$, CEMTC-11036$_{K64}$, CEMTC-11037$_{K64}$, CEMTC-11038$_{K64}$, H$_{K64}$,and CEMTC-11041$_{K2}$

Serial tenfold dilutions (5 µl) of each phage were spotted onto a top agar lawn of exponentially growing *Klebsiella* host strains. The phage stock concentrations for KP192_tspA195 was 1 × 10^{10} PFU/mL. For KP192_tspN$_{192}$A195, an eluate from several plaques was used, with an approximate concentration of 1 × 10^{5} PFU/mL. Assays were performed on 90 mm Petri dishes; representative 10 × 10 mm areas are shown at higher magnification.

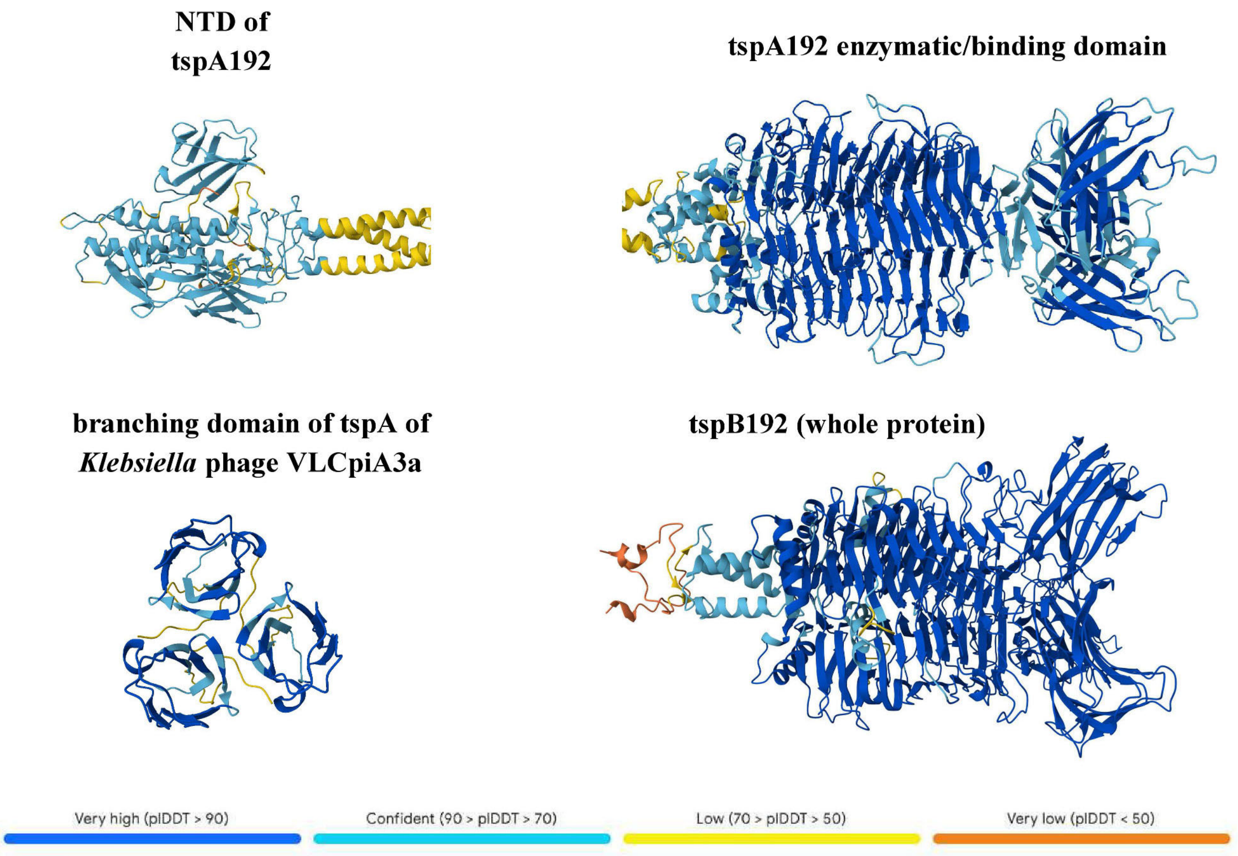

Figure S5. AlphaFold3 models of functional domains in tspA192 and tspB192 proteins

The branching domain from the related phage, VLCpiA3a, was used as a structural template for the corresponding unmodeled region in tspA192. Models are colored by predicted local distance difference test (pLDDT) confidence score.

Table S1. Primers used for PCR-amplification of fragments of phage genomes, related to Figure 2B and "Preparation of DNA Fragments for Assembly of Phage Genomes" method.

Primer name	Sequence
pRSII415_192/5_genome_dir	5'GATACTATATGTTGATGTCTCTGTGTCCCTTGTCTCATGAGCGGATACATA3'
pRSII415_192/5_genome_rev	5'GGGGGATAACCAAAAGTGTAAACTGTGAGACCTTGTTCATGTGTGTTCAAAAAC3'
pt1_dir	5'TCTCACAGTTTACACTTTTGGT3'
pt1_rev	5'CATTCGAAGGTTCCCTATAG3'
pt2_dir	5'GCTGGGAATCTCTTTAAGGC3'
pt2_rev	5'CACCTACGTCCATGGTTGCCT3'
pt3_dir	5'GGTTAGTGTTCTCTTCGAGTCGC3'
pt3_rev	5'GCTGAGAAGTCCCAAGGTCG3'
pt4_dir	5'GCAGACATCGAGAAGCTGACC3'
pt4_rev	5'GCGCACAGGACTCTGCTCG3'
pt5_dir	5'GTCGTGGAGGACGTTGAGTC3'
pt5_rev	5'GGACCTGATACTCATAGCCGG3'
pt6_dir	5'GCCTGAAATCCTACGGTACCC3'
pt6_rev	5'AGTAGCTCCTTGTCGTAGCGG3'
pt7_dir	5'GACGTGGATGGTGATACCATTG3'
192_pt7_rev	5'ACCTCCTTTAGTTGGATGAGAAG3'
195_pt7_rev	5'GCCTCCTTAAGTTGAATGTGGAG3'
192_pt8_dir	5'CAGCCGAAGCCTAACTAATTAGG3'
195_pt8_dir	5'CAGCAGCCTAAACCTAACTAATT3'
pt8A_tf192.N150+tfA195.C_rev	5' GTCAGCGATTATCTTGTTGAGTTGATTCTTGTTGACAGCGTCTGT 3'
pt8A_tf195.N150+tfA192.C_rev	5' CTCACCTAAGGTTGTGTCCAGCTGCTGAACGGTTACTGCAT 3'
pt8B_tf195.N150+tfA192.C_dir	5' GATGCAGTAACCGTTCAGCAGCTGGACACAACCTTAGGTGAG 3'
pt8B_tf192.N150+tfA195.C_dir	5' ACAGACGCTGTCAACAAGAATCAACTCAACAAGATAATCGCTGACG 3'
pt8_rev	5'TCAGCCCCAGCGACCCCCG3'
pt9_dir	5'TATCCCTAGACTTCAACAACGAAG3'
pt9_rev	5'AGGGACACAGAGACATCAACATA3'

Table S2. Genomic DNA fragments used for phage genomes assembly and corresponding primers used for their amplification, related to Figure 2B and "Preparation of DNA Fragments for Assembly of Phage Genomes" method.

**Fragment name**	**Primers used for amplification**	**Template DNA**
Fragments of KP192 phage genome		
192_pt1	pt1_dir + pt1_rev	KP192 genomic DNA
192_pt2	pt2_dir + pt2_rev	
192_pt3	pt3_dir + pt3_rev	
192_pt4	pt4_dir + pt4_rev	
192_pt5	pt5_dir + pt5_rev	
192_pt6	pt6_dir + pt6_rev	
192_pt7	pt7_dir + 192_pt7_rev	
192_pt7+8A [1]	pt7_dir + "pt8A_tf192.N150+tfA195.C_rev"	
192_pt8	192_pt8_dir + pt8_rev	
192_pt8B	"pt8B_tf195.N150+tfA192.C_dir" + pt8_rev	
192_pt9	pt9_dir + pt9_rev	
Fragments of KP195 phage genome		
195_pt1	pt1_dir + pt1_rev	KP195 genomic DNA
195_pt2	pt2_dir + pt2_rev	
195_pt3	pt3_dir + pt3_rev	
195_pt4	pt4_dir + pt4_rev	
195_pt5	pt5_dir + pt5_rev	
195_pt6	pt6_dir + pt6_rev	
195_pt7	pt7_dir + 195_pt7_rev	
195_pt7+8A	pt7_dir + "pt8A_tf195.N150+tfA192.C_rev"	
195_pt8	195_pt8_dir + pt8_rev	
195_pt8B	"pt8B_tf192.N150+tfA195.C_dir" + pt8_rev	
195_pt9	pt9_dir + pt9_rev	
vector fragment	pRSII415_192/5_genome_dir + pRSII415_192/5_genome_rev	pRSII-415 plasmid

[1.] The fragments indicated in italics and blue were used for the assembly of phages with chimeric type A tailspikes.

Table S3. DNA fragment combinations used for assembly of phage genomes, related to Figure 2 and "Phage Genome Assembly in Yeast" method.

**Fragment name**	**KP192ctrl**	**KP192_tsp N_{192}A195**	**KP195ctrl**	**KP195_tsp N_{195}AB192**
**Fragments of KP192 phage genome**				
192_pt1	+	+		
192_pt2	+	+		
192_pt3	+	+		
192_pt4	+	+		
192_pt5	+	+		
192_pt6	+	+		
192_pt7	+			
192_pt7+8A [1]		+		
192_pt8	+			
192_pt8B				+
192_pt9	+	+		
**Fragments of KP195 phage genome**				
195_pt1			+	+
195_pt2			+	+
195_pt3			+	+
195_pt4			+	+
195_pt5			+	+
195_pt6			+	+
195_pt7			+	
195_pt7+8A				+
195_pt8			+	
195_pt8B		+		
195_pt9			+	+
**Vector fragment**	+	+	+	+

1. The fragments indicated in italics and blue were used for the assembly of phages with chimeric type A tailspikes.

Table S4. Primers used for Sanger sequencing and phage sample validation, related to "Verification of Genome Assembly Accuracy" method.

**Primer name**	**Sequence**
KP192_10950bp_valid_dir	5'TCTGTACAAATTATGGTCAGGCTAA3'
KP192_11110bp_valid_rev	5'ACCTACCTCGTGTAATACACCGATT3'
KP192_tspB_36450bp_valid_dir	5'CTTCTCAGACCAGACCGCTTGTGCT3'
KP192_tspB_37050bp_valid_rev	5'CACGAACACGGAGAGATATCTCA3'
KP195_9930bp_valid_dir	5'GTCGTCTGAATCCTGAAGCACT3'
KP195_10320bp_valid_rev	5'CTGCACCTCTCTTAATGTTG3'
KP195_tsp_36750_valid_dir	5'TGGTCCAACTGGAGCGAGTGCC3'
KP195_tsp_37000bp_valid_rev	5'TTCAGGTACACTCCGGTGATTGT3'
pt7_ivpD192/5_seq_dir	5'AAGGAGCTTGTTCCGAACGACCC3'
pt8_tsp195_seq_v2_rev	5'GCTACCGTCAACAAGCGTGATTCCG3'
